# Supplementary material for: A novel system for classifying tooth root phenotypes
Source: PLoS One. 2021 Nov 5;16(11):e0251953. doi: 10.1371/journal.pone.0251953 (PMC8570528; doi:10.1371/journal.pone.0251953)
Supplement: S2 Table — (DOCX) [file pone.0251953.s002.docx]

**S2: Observer error test**

James Clark, PhD student at the University of Cambridge Leverhulme Centre for Human Evolutionary Studies agreed to analyze CT scans of the below individuals and classify their tooth root phenotypes using the methods outlined in the study. Below are his results compared to lead author Jason Gellis.

ID: AF_15_0_47 Maxilla

| Tooth | James Clark | Jason Gellis | Accuracy |
| --- | --- | --- | --- |
| P^3^ | 2_2_B1L1_BKLG_BRLR | 2_2_B1L1_BKLG_BRLR | 100.00 |
| P^4^ | 2_2_B1L1_BKLG_BRLR | 2_2_B1L1_BKLG_BRLR | 100.00 |
| M^1^ | 3_4_M2D1L1_MWDPLE_MR2D[R]LO | 3_4_M2D1L1_MWDPLE_MR2D[O]LO | **91.00** |
| M^2^ | 3_4_M2D1L1_MWDPLE_MR2DRLO | 3_4_M2D1L1_MWDPLE_MR2DRLO | 100.00 |
| M^3^ | 3_3_M1D1L1_MWDELG_MODRLR | 3_3_M1D1L1_MWDELG_MODRLR | 100.00 |

[ ] = mismatch

ID: AF_15_0_55 Maxilla

| Tooth | James Clark | Jason Gellis | Accuracy |
| --- | --- | --- | --- |
| P^3^ | 2_2_B1L1 _BGLG_BRLR | 2_2_B1L1_BGLG_BRLR | 100.00 |
| P^4^ | 2_2_B1L1_BGLG_BRLR | 2_2_B1L1_BGLG_BRLR | 100.00 |
| M^1^ | 3_4_M2D1L1_MWD[E]LE_MR2DRLR | 3_4_M2D1L1_MWDPLE_MR2DRLR | **91.00** |
| M^2^ | 3_4_M2D1L1_MWDELG_MR5DOLR | 3_4_M2D1L1_MKDELG_MR5DRLR | 100.00 |
| M^3^ | 3_3_M1D1L1_MKDGLG_MRDRLR | 3_3_M1D1L1_MKDGLG_MRDRLR | 100.00 |

[ ] = mismatch

ID: AF_15_0_62 Maxilla

| Tooth | James Clark | Jason Gellis | Accuracy |
| --- | --- | --- | --- |
| P^3^ | 2_2_B1L1_BKLE_BRLR | 2_2_B1L1_BKLG_BRLR | 100.00 |
| P^4^ | 2_2_B1L1_BKLE_BRLR | 2_2_B1L1_BKLG_BRLR | 100.00 |
| M^1^ | 3_3_M1D1L1_MWDPLP_MRDRLO | 3_3_M1D1L1_MWDPLP_MRDRLO | 100.00 |
| M^2^ | 3_4_M2D1L1_MWDELE_MR2DRLR | 3_4_M2D1L1_MWDELE_MR2DRLR | 100.00 |
| M^3^ | 3_3_M1D1L1_MWDGLG_MRDRLR | 3_3_M1D1L1_MWDGLG_MRDRLR | 100.00 |

[ ] = mismatch

ID: AF_15_0_64 Maxilla

| Tooth | James Clark | Jason Gellis | Accuracy |
| --- | --- | --- | --- |
| P^3^ | 2_2_B1L1_BGLG_BRLR | 2_2_B1L1_BGLG_BRLR | 100.00 |
| P^4^ | NA | NA | NA |
| M^1^ | 3_4_M2D1L1_MHDPLP_MR4DRLO | 3_4_M2D1L1_MWDPLP_MR4DRLO | 100.00 |
| M^2^ | 3_4_M2D1L1_MHDELE_MR4DRLO | 3_4_M2D1L1_MHDELE_MR4DRLO | 100.0 |
| M^3^ | 3_3_M1D1L1_MWDGLG_MRDRLR | 3_3_M1D1L1_MWDGLG_MRDRLR | 100.0 |

[ ] = mismatch

ID: AF_15_0_64 MANDIBLE

| Tooth | James Clark | Jason Gellis | Accuracy |
| --- | --- | --- | --- |
| P_3_ | NA |  |  |
| P_4_ | NA |  |  |
| M_1_ | 2_5_M2D3_MHDP_MR2Di4 | 2_5_M2D3_MHDP_MR2Di4 | 100.00 |
| M_2_ | 2_4_M2D2_MHDK_MR4Di2 | 2_4_M2D2_MHDK_MR4Di2 | 100.00 |
| M_3_ | 2_3_M2D1_MKDP_MR4DO | 2_3_M2D1_MKDP_MR4DO | 100.0 |

[ ] = mismatch
