# Supplementary material for: A novel system for classifying tooth root phenotypes
Source: PLoS One. 2021 Nov 5;16(11):e0251953. doi: 10.1371/journal.pone.0251953 (PMC8570528; doi:10.1371/journal.pone.0251953)
Supplement: Attachment — Submitted filename: response_to_reviewers.docx [file pone.0251953.s003.docx]

Dear Dr. Schepartz, and Reviewers 1 & 2,

Thank you for your response to our submission, and for the helpful and constructive comments of the reviewers. We have addressed all their concerns and made corrections as necessary. These are summarised in the Table below. We note that you asked us in particular to give attention to -

1. The concerns of the academic editor – *please see the Table below, where the comments are categorised by Reviewer.*

2. The comments of Reviewer 1 - *this we have done, see the Table below, where the comments are categorised by Reviewer.*

3. The observer error is important for readers to assess the ease in using the system - *we have now carried out an observer error check, and this is reported in the text, lines 295 -298, with supporting information in the Supplementary information. The results showed that the method is replicable.*

4. The slice thickness query of Reviewer 2. - *this has been addressed (see Table), and a further explanation added to the text (lines ….)*

5. Moving the anatomical description section as suggested by Reviewer 2. - *this has been done.*

6. The minimal anonymized data set necessary to replicate this study’s findings is now available at the Open Science Framework –

- https://osf.io/9ynur/
- DOI 10.17605/OSF.IO/9YNUR

We hope these changes are acceptable and look forward to hearing from you. If you require further information, please let us know.

Yours sincerely,

Dr. Jason Jacob Gellis & Professor Robert Andrew Foley

| **Academic editor** |  |
| --- | --- |
| p. 3 line 52 'a world frequency' does not have a range; rephrase | This has been rephrased to - *Scott and Turner [2] report that Sub-Saharan Africans have the highest frequency at 65%, 40% in West Eurasian populations, 20-30% in East Asian populations, and 5-15% in Northeast Siberians and Native Americans.* Lines 52-54 |
| p. 19, line 415: the first 'their' should be 'therefore'. | This has been corrected to ‘therefore’ |
| p. 21, line 449 has grammatical errors and missing words; rephrase | Rephrased to - *As discussed in the literature review above, the categorization of roots and canals can be misleading or inaccurate when systems devised for a particular tooth type (e.g. premolar) are applied across other tooth types.* - lines 529 -530 in the revised manuscript. |
| p, 25 line 584 phenotype permutations, not 'phenotypes' | Corrected to - *…how permutations in one phenotypic element can result in...* Lines 659-660 in the revised manuscript. |
| Rename 2^nd^ table 10 to table 11 | Corrected to Table 11 in the revised manuscript. |
| Please include captions for *all* your Supporting Information files at the end of your manuscript, and update any in-text citations to match accordingly. | These have been added/corrected according to guidelines provided by PLOS ONE. |
| **Reviewer 1** |  |
| I’m worried that the orientation of each tooth, although the jaw was placed in an anatomical position, might affect the score of external and internal morphologies. For example, a round canal cross-section might be observed in an elliptical shape if an oblique slice was obtained. A completely quantitative method, in 3D context, might overcome all the problems of qualitative scoring system. | This problem has been addressed in the new section titled ‘Exclusion Criteria’ (see lines 286-294). The relevant section is quoted here -  *Skulls and/or mandibles that were misaligned/poorly aligned to the anatomical plane might distort morphologies (e.g., a round canal may appear oval shaped if the CT slice is at an oblique angle). This problem is mitigated by visualizing the morphology in question across multiple planes through the CT stack, and as a CT volume. However, for cases in which the researcher felt unsure, these individuals were excluded from analysis.* |
| In the legend of Figure 3, which level did the author get the cross-section? Indicate what "E, G, H, and P" means in the figure legend. | E, G, H, and P have been defined as – elliptical (E), globular (G), hourglass (H), and plate (P), in the revised manuscript. |
| Figure 4 and 9 are the same, except for the labels. | While these figures are nearly identical, the first is used to describe morphology, while the second is used to illustrate how these morphologies are labelled. |
| **Reviewer 2** |  |
| In general, there are a significant number of figures, which ideally could be condensed to reflect the most pertinent figures in the study. | While we agree that there are a large number of figures, we feel the number is appropriate for the study. |
| As this is a scientific article all references to "we" should be removed, | This has been corrected throughout the manuscript. |
| The introduction would benefit from including a short section discussing the potential genetic abnormalities associated with root morphology, particularly considering that the "normal" morphology of the roots is the subject under investigation. | We recognise the reviewer’s concern that there are pathological abnormalities, often associated with particular genes, but as we have excluded these from the sample (see new ‘exclusion criteria’ section on line 286) we did not think it appropriate to extend the introduction on this topic as it lay outside the aims of the paper. We have made the point more explicitly in the ‘Exclusion Criteria’. |
| Pg. 3, line 47, reference numbers can be condensed to reflect 12-26. | This has been corrected to [12-26] in the revised manuscript. |
| In the legend for figure 1, line 77, specify which molars are being referenced to keep consistency with the rest of the legend. | The first line of the figure has been changed to Mandibular 1^st^ molars, while the remaining labels use M_1_ |
| Pg. 5, Line 124, replace "Though" with "While". | This has been corrected to ‘while’ in the revised manuscript. |
| Line 127, insert references, particularly if you are talking about descriptions which appear in the literature. | References have been added where appropriate. |
| Please change specimens to individuals throughout. | Specimens have been changed to individuals throughout the revised manuscript. |
| Do you think the difference in slice thickness may have resulted in a loss of observed detail in the scans? | We have addressed this issue explicitly in lines 244-247. |
| No exclusion criteria are mentioned and should be included. | A section outlining exclusion criteria has been added to the materials and methods section. Some aspects of this are also discussed in the discussion section |
| The tenses in this section need revision. As the article happens after the study the article should be written in the past tense. Please correct throughout. | This has been corrected throughout the revised manuscript. |
| Pg. 11. line 312, Anatomical descriptions should come earlier, preferably straight after the CT section. | This section has been moved according to recommendation. |
| No mention of observer error is mentioned at any point, this is problematic considering the proposal of novel system for classifying teeth. | Observer error test is mentioned in lines … of the revised manuscript. The test itself is now in the supplementary materials. |
| Results: Pg. 12 Lines 331 to 348 should ideally come under the materials and methods as it is an admixture of a sample description as well as data analysis. | We can see that these lines are a combination of description and analysis, but we think that the analysis element is the dominant part, and the description necessary to make the analysis understandable. As such we would prefer to leave this in the results section where it will be more visible to the reader, as it is an important element of our findings. |
| The redundancy of information section, line 547 should not be in the results section and ideally should form part of the discussion section. | This section has been moved according to recommendation. |
| The results section requires extensive revision given that across various points in the results, i.e., Table 6 line 404, there appears to be a mixture of result reporting and discussion of the results. | We think this change would be detrimental to the clarity of the paper, and would argue that there is minimal ‘discussion’. For example, in the example Reviewer 2 refers to Table 6 , but this is simply a description of the external root morphologies our study has  shown (a key result), and the text related to Table 6 is a minimal support for the table findings. |
| There is a figure in the discussion which is unusual. | We think that it is appropriate in this context to add the figure as it  clarifies and summarises the discussion of the results, and places them in the broader context of the thinking about phenotypic diversity. |
